# Using Genetic Networks and Homology to Understand the Evolution of Phenotypic Traits

**DOI:** 10.2174/138920212799034785

**Published:** 2012-03

**Authors:** Amy R McCune, John C Schimenti

**Affiliations:** 1 Department of Ecology and Evolutionary Biology, Corson Hall, Cornell University, Ithaca, NY 14853, USA; 2 Department of Biomedical Sciences, Cornell University, Ithaca, NY 14853, USA

**Keywords:** Gene regulatory networks, genomics, deep homology, taxic homology, transformational homology, Next Gen Sequencing.

## Abstract

Homology can have different meanings for different kinds of biologists. A phylogenetic view holds that homology, defined by common ancestry, is rigorously identified through phylogenetic analysis. Such homologies are taxic homologies (=synapomorphies). A second interpretation, “biological homology” emphasizes common ancestry through the continuity of genetic information underlying phenotypic traits, and is favored by some developmental geneticists. A third kind of homology, deep homology, was recently defined as “the sharing of the genetic regulatory apparatus used to build morphologically and phylogenetically disparate features.” Here we explain the commonality among these three versions of homology. We argue that biological homology, as evidenced by a conserved gene regulatory network giving a trait its “essential identity” (a Character Identity Network or “ChIN”) must also be a taxic homology. In cases where a phenotypic trait has been modified over the course of evolution such that homology (taxic) is obscured (e.g. jaws are modified gill arches), a shared underlying ChIN provides evidence of this transformation. Deep homologies, where molecular and cellular components of a phenotypic trait precede the trait itself (are phylogenetically deep relative to the trait), are also taxic homologies, undisguised. Deep homologies inspire particular interest for understanding the evolutionary assembly of phenotypic traits. Mapping these deeply homologous building blocks on a phylogeny reveals the sequential steps leading to the origin of phenotypic novelties. Finally, we discuss how new genomic technologies will revolutionize the comparative genomic study of non-model organisms in a phylogenetic context, necessary to understand the evolution of phenotypic traits.

## 1. INTRODUCTION

The Genome Project in the 1990s and early 2000s was conducted in a very different technological era from today. Through the 20th century, the discipline of Genetics was essentially limited to a few model organisms –such as *Escherichia coli, Saccharomyces cerevisiae, Drosophila melanogaster, *and* Mus musculus* - around which biological and molecular infrastructure were accumulated over decades (such as mutations; specialized strains; genetic markers). These resources, coupled with both molecular biology tools that were developed in the latter quarter of the century, and the appearance of the first generations of automated DNA sequencers, enabled the genomic mapping and sequencing of these several organisms, plus humans. The cost of generating the first draft human genome sequence was ~ $300 million dollars. Absent the benefits of a genome sequence and other genetic resources, pioneers in the study of evolutionary (comparative) developmental biology were limited to studies of individual genes and gene families (such as *Hox*, *Distaless*, *Pax*), the structures and expression of which had to be elucidated painstakingly in organisms of interest.

With the advent of “Next-Generation Sequencing” (NGS) technologies and genomic analysis methods, the scope for genomic studies of non-model organisms has expanded dramatically. What is truly exciting for both molecular genetics and evolutionary biology is that these modern methods greatly enhance our ability to uncover the genetic basis of the evolutionary transformation of phenotypic traits in virtually any non-model organism. Critical to understanding evolutionary transformation of traits is use of a rigorous phylogenetic framework (evolutionary tree), to specify the scope, direction, and sequential steps of evolutionary change. The key concept allowing comparative biologists to reconstruct a phylogenetic framework (tree of life) is homology, that is, similarity due to common ancestry. Homology is fundamental to understanding the evolution of phenotypes, that is, how evolution has modified development over time to create the vast diversity of life. Homology is also the reason that what is learned by the study of model organisms has generality in biology. Given the pervasive importance of homology for understanding biology and evolution, we review here how homology has been used differently by different kinds of biologists and show the commonality of these varied concepts of homology. We then discuss how the study of homology in a phylogenetic framework can reveal the sequential steps leading to the origin of important evolutionary novelties, such as jaws or limbs, that differ qualitatively from their antecedents (i.e., gill arches or fins respectively). And finally we discuss how the newest advances in genomic technology will further enhance our ability to expose the genetic basis for evolutionary change in (model and non-model) organisms.

## 2. IMPLICIT AND EXPLICIT USES OF HOMOLOGY

Using a model organism to learn about the molecular basis of human disease or to test a treatment depends on the organs or metabolic pathways being homologous. Often the best models for studying human biology are our closest relatives (e.g. primates or mammals). However, some organs, pathways or functions are more conserved than others; the more conserved the biological attribute, the greater the phylogenetic breadth of potentially relevant model organisms. For example, to the extent that mechanisms of gene regulation are highly conserved across eukaryotes, studies in fruit flies can be generalized to other eukaryotes. However, the liver is unique to vertebrates so studies of liver function must be done on a vertebrate; how conserved liver function is across vertebrates will affect the utility of various vertebrate models for understanding human liver function. For less conserved aspects, study of mammalian or primate liver function may be most informative. For more conserved aspects, it might be that any vertebrate model will do. 

The idea that the taxonomic inclusiveness or phylogenetic level (e.g. there is increasing inclusiveness or a deeper phylogenetic level from primates to mammals to vertebrates to animals, to eukaryotes) at which a biological attribute is “conserved” can be specified is the fundamental idea that has allowed evolutionary biologists to reconstruct the tree of life. If a trait, like liver formation, is unique to a group (clade) like vertebrates, we would say that the liver is a homology of vertebrates. No one would disagree with the statement that the livers of mice and humans are homologous. However, there have been other uses of the term homology, some of which have been used (and sometimes misused) by every kind of biologist. Homology is such a fundamental concept for comparative biology, it is not surprising that the concept has been the subject of much controversy, discussion and debate (e.g. [1-3]).

## 3. ALTERNATIVE VIEWS OF HOMOLOGY

In the last 30 years, two alternative ways of thinking about homology have solidified and are being used by different kinds of biologists. One, phylogenetic or “taxic” homology, was crystallized by Patterson [4] and is used by systematists and many evolutionary biologists. Patterson recognized that homologies are equivalent to “synapomorphies.” *Synapomorphies or taxic homologues are shared derived characters, similar due to common ancestry*. Any characteristic of a taxon, from SNPs to gene networks, to morphologies or behaviors, can be a taxic homology. Synapomorphies (=homologies) define natural groups of organisms (taxa) and nested distributions of such characters are used to reconstruct phylogenies. Similarity *due to common ancestry* is a key idea because derived characters shared by two or more taxa can also be similar due to convergence, as exemplified by the wings of insects and bats; no one would use wings to define a group of animals because wings are known to have arisen independently in birds, bats, and insects. However, within the more limited phylogenetic context of insects, wings are a synapomorphy or homology of the taxon, “winged insects” (=Pterygota). Likewise, having vertebrae is a homology or synapomorphy of vertebrates; having limbs is a homology of tetrapods (4-footed vertebrates); having segments is a homology of arthropods. Orthology, or similarity of DNA sequence in two or more taxa due to speciation (not gene duplication) is homology in this taxic or phylogenetic sense. A taxic homologue is most rigorously identified with respect to the group it characterizes: vertebrae are a homology of the group Vertebrata; [3, 4]; *Pax6* is a homology (or conserved at the level) of Bilateria. While this terminology was first used in a morphological context, it is just as powerful for specifying the evolutionary history of any biological trait at any biological level of organization (e.g., genetic, cellular, developmental, physiological, behavioral, etc.).

Another concept of homology, known as “biological homology,” has been favored by some developmentally-oriented biologists. It emphasizes the *historical continuity of genes *underlying the development of phenotypic characters [5-7]. After all, the reason that all vertebrates have livers and all arthropods have segments is because of an underlying shared genetic basis for making liver in the vertebrate lineage and an underlying shared genetic basis for making segments in arthropods. Still, as intuitive as this idea seems, in the last two decades, developmental genetics has demonstrated repeatedly the “apparently loose relationship between morphological characters and their genetic basis” [8] making it difficult to apply the concept of biological homology. Just because *Pax6* is involved in the development of both vertebrate and cephalopod eyes it does not mean that vertebrate and cephalopod eyes are homologous. In fact, we know that the gene, *Pax6,* is older than the most recent common ancestor of vertebrates and cephalopods and that it was co-opted independently in these two groups [8-10]. The challenge for proponents of biological homology is that, despite the continuity of genetic information (*Pax6*) in the development of both vertebrate and cephalopod eyes, vertebrate and cephalopod eyes are not homologous because the gene was co-opted independently in eye development in the two groups. We know this because a phylogenetic analysis of animal phyla shows that the common ancestor of molluscs and vertebrates did not have camera eyes. Consistent with this phylogenetic result is that the development and structure of cephalopods and vertebrate eyes differ in detail. Thus, an evolutionary biologist would recognize the gene *Pax6* as an homology of Bilateria (conserved across bilaterians). Nested within bilaterians, vertebrate eyes are a homology of vertebrates and cephalopod eyes are a homology of cephalopods (within molluscs) but *Pax6 *was co-opted in eye development independently in these two groups or taxa. The point is that a gene, like *Pax6*, can characterize a much more inclusive group (e.g., Bilateria) than some of the structures it is involved in specifying (e.g. vertebrate eyes) and the same gene can be involved in specifying multiple structures, structures that themselves are not homologous (e.g. vertebrate eyes and cephalopod eyes). The fact that the origin of genes or cell types can precede the origin of phenotypes that incorporate them has been termed *deep homology *[10]. Deep homologies are a special kind of taxic homology. Their importance is that deeply homologous molecular and cellular components (whether it be a gene, cell type, signaling pathway or gene regulatory network) contributing to phenotypic novelties enable us to reconstruct how a phenotype was built over evolutionary time.

The conundrum for proponents of biological homology is that we perceive that the developmental genetic underpinnings of a phenotypic feature ought to be evidence for the common origin of phenotypic features; yet when genes have origins that precede the features of interest, underlying genetics does not provide evidence of common ancestry of structures, that is, of homology. To solve this conundrum, one of the principal advocates of biological homology [8] has suggested that, while single genes cannot provide convincing evidence of common ancestry, a gene regulatory network (GRN) *can*. Conceptually, this is the same idea that complex morphological traits (complex meaning more components, more interactions) provide better evidence of homology than do simple morphological traits. Wagner has further suggested that homologous morphological features are underlain by a core network of developmental genes, giving those features their “essential character.” He terms this core gene network a “Character Identity Network” or “ChIN” [8]. Such core gene networks would be necessary for the development of a trait, but probably not sufficient.

Building on both Patterson’s [4], Shubin *et al.*’s [10] and Wagner’s [8] insights, we argue: (1) that a potential “character identity network” [ChIN] must also be a taxic homology, identified using phylogenetic methods (implicitly or explicitly); (2) once a gene network has been identified as a taxic homologue and a ChIN, study of that gene network provides a basis for analyzing character evolution (transformation) at both the phenotypic and genotypic levels; (3) specifying deep homologies, that is, identifying the phylogenetic levels at which various molecular and cellular components of a phenotype have arisen (are homologous) allows us to reconstruct the sequence by which evolution has built novel phenotypes, and (4) advances in comparative genomics of non-model organisms will facilitate identification of gene networks and functional analyses of network interactions critical to understanding how a morphological phenotype is specified and has been modified through the course of evolution.

### 3.1. Phylogenetic or Taxic Homology

**Fig. (1).**
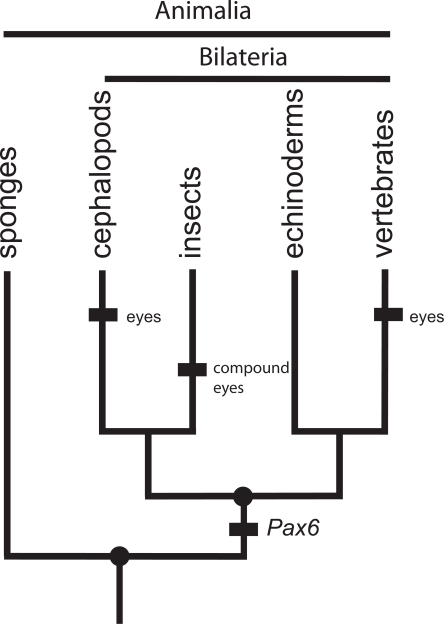
**Homologous genes do not always make homologous structures.** *Pax6* is an ancient gene, homologous at least at the level of Bilateria. Following its origin, *Pax6* was co-opted to become involved in the development of a number of different structures in different lineages. Just because *Pax6* is involved in the development of both insect compound eyes and vertebrate eyes, does not mean that insect and vertebrate eyes are homologous. In the context of a phylogeny, we can see that the origin of *Pax6* preceded the origin of eyes in both lineages and must have been independently co-opted. *Pax6* and other genetic and cellular components of eyes that have arisen before eyes are deep homologies [10].

To have an operational concept of homology, biologists on all sides of the debate will sometimes resort to a common, rough approximate of a definition: homology is similarity due to common ancestry. On the phylogenetic side, Patterson’s insight [4] was to recognize that homology is equivalent to the concept of synapomorphy (shared derived character due to common ancestry), because *every feature we might want to label as homologous is a synapomorphy at some level in the phylogenetic hierarchy*. Vertebrae of rodents and bats are homologous because vertebrae are a synapomorphy characterizing the group Vertebrata. Rigorous statements of homology include a conditional phrase specifying the nature of the trait *and* the phylogenetic level: wings of birds and bats are homologous *as forelimbs of tetrapods*, where forelimbs specify the trait and tetrapods specify the level (note that wings of birds and bats are not homologous as wings at any level) [3, 4]. Taxic homologies can be features at any level of biological organization: from a complex phenotypic trait like jaws, to a gene, to a gene expression pattern, to a nucleotide substitution [9, 11] or a complex genotypic trait like a gene regulatory network, as long as it is a trait which characterizes a natural group (i.e. a monophyletic group comprising the common ancestor and *all* its descendants) at some level in the phylogenetic hierarchy. The GRN specifying neural crest is likely a taxic homology of chordates and the GRN specifying eye field in arthropods is likely a taxic homology of arthropods [12]. However, the gene *Pax6*, involved in the eye specification GRN, is conserved at a much deeper taxonomic level than arthropods, perhaps the Bilateria. That is, *Pax6* is likely an homology of Bilateria (Fig. **1**).

Homologies, that is, taxic homologies, are characters that define natural (monophyletic) groups; homology is synonomous with synapomorphy. Given the utility and pervasiveness of tree thinking [13] in modern biology, irrespective of whether traits are molecular or morphological, this concept of taxic homology has power and persistence. It is *the* fundamental concept allowing us to reconstruct the history of life and homology underlies any study of phenotypic or genotypic transformation.

### 3.2. Biological Homology and Character Identity Networks (ChINs)

**Fig. (2).**
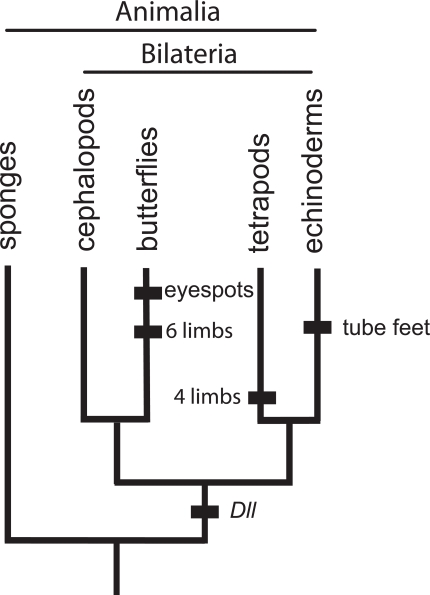
**Homologous genes can be deployed in completely distinctive, non-homologous structures.** *Distal-less* is a taxic homology of at least the Bilateria; it is conserved across Bilateria. But *Distal-less* has been co-opted independently in the development of eyespots in butterflies, tetrapod limbs and tube feet of echinoderms. These three structures are not homologous.

Proponents of biological homology have emphasized the underlying developmental and genetic basis for traits of organisms. After all, the reason that having vertebrae characterizes the group Vertebrata is that a developmental genetic system making vertebrae, albeit with various modifications, has been passed down from ancestor to descendent for more than 500 million years. In this view, it is “continuity of information” that causes homology [5, 6, 14]. Still, a main proponent of the biological definition of homology argued in a recent review that the concept of [biological] homology remains “highly elusive” because of “the apparently loose relationship between morphological characters and their genetic basis” [8]. It is well-known that a gene can be co-opted and deployed in non-homologous features—the gene, *Distal-less (Dll),* is involved in the development of butterfly eyespots, starfish tube feet, and tetrapod limbs, but no one would suggest that butterfly eyespots and tetrapod limbs are homologous (Fig. **2**). This loose relationship, well known to de Beer [15] and Roth [7, 14], is the reason that homology has been so “disappointing and confusing” to proponents of biological homology ([8], p.474). Aboueif [9] attempted to integrate the “phylogenetic” and “biological” approaches to account for this problem by arguing that the homologies of genes, embryonic patterns of development, gene expression, and morphology needed to be considered separately in a phylogenetic context. This approach formalizes the utility of specifying that a gene, such as *Distal-less, *can be a taxic homology of Bilateria (that is, conserved across bilaterians) while its deployment in a phenotypic trait, such as tetrapod limbs can be a taxic homology of a much more restricted clade, in this case, tetrapods.

Wagner [8] has suggested a solution to the loose relationship between morphology and the underlying genetics within the biological homology framework: “the continuity of morphological characters could be underwritten by homologous regulatory networks of co-adapted transcription factor genes, whereas other aspects of their development can be variable.” Homologous regulatory network means that the regulatory networks in different taxa would have been “derived from the same network in their most recent common ancestor” [8]. Thus, while variable aspects of development can make the relationship between morphological homologies and their underlying development seem loose, Wagner suggests that morphological homologies have, at their developmental core, a highly conserved gene regulatory network that gives the morphological homology its “essential character.” Such highly conserved gene networks have previously been termed “kernels” [12]. Wagner has suggested that the kernel responsible for a phenotypic character’s essential identity, say causing forewing versus hindwing development, be termed a “character identity network” or “ChIN”.

## 4. SYNTHESIS: USING HOMOLOGY TO UNDERSTAND HOW PHENOTYPES EVOLVE

### 4.1. ChINs are taxic Homologies

How might “ChINs” be related to the phenotypic homologies they underlie? That is, what is the relationship between taxic homology as exemplified by a phenotypic character (say, segmentation in arthropods) and its underlying “ChIN,” a gene network specifying the essential nature of segmentation? How does one identify a “ChIN?”

Characterizing gene expression and the interactions of genes in a network is clearly the exclusive purview of developmental genetics and functional genomics. But once a genetic network has been characterized, identification of that network as a conserved kernel and a ChIN must involve comparative study and phylogenetics. As defined by Davidson and Erwin [12], kernels must be highly conserved and invariant across the members of the group being compared; that is a kernel is an homology of a particular group or clade. For a kernel to be a ChIN, it must be shown that (1) the kernel is conserved at the same phylogenetic level as the phenotypic trait it is hypothesized to underlie, and (2) the core conserved network is responsible for the “essential identity” of the phenotypic trait of interest. The first criterion can be satisfied with phylogenetic analysis while the latter obviously requires experimental manipulation.

In developing the ChIN concept, Wagner suggests that the segment polarity network may be a ChIN for insect segmentation because it is “invariant, at least among insects,” in contrast to the gap genes and pair-rule genes which are variable among insects [8]. Thus, “the most conservative parts of the developmental process are the GRNs that control the developmental programme that specifies the identity of the character; that is, the character identity network (ChIN).” If both the segment polarity network and morphological segmentation are found to be conserved across insects (or perhaps the more inclusive group, arthropods), but not across other lineages related to arthropods, the segment polarity GRN would be a taxic homology of arthropods and a good candidate for a segmentation ChIN. If the segment polarity network is homologous (conserved) at a deeper phylogenetic level, then it cannot be an arthropod segmentation ChIN, but it could be a ChIN for some phenotypic trait of a more inclusive group. In others words, a ChIN must first be a taxic homology. The phylogenetic group across which the network is conserved specifies the level at which the network is homologous. The fact that some segment polarity genes are also found in mammals could be explained either by the network being homologous at deeper level (e.g., Bilateria) or it could be that these component genes have been co-opted independently into two different networks, one in arthropods and the other in mammals. Only comparative studies of gene networks can differentiate between these two hypotheses. In fact, the long-rejected hypothesis that segments in vertebrates and arthropods are homologous [16] has recently been resurrected and is the subject of current debate, motivating careful comparative mechanistic studies of gene expression in pertinent non-model organisms [17]. It remains to be seen whether there is a segmentation ChIN unique to arthropods, but for this and every other hypothesized ChIN, a phylogenetic framework is key to specifying the level of homology (i.e. an homology is conserved for a particular group but not a more inclusive group).

A case where a GRN is not validated as a taxic homology is eye morphogenesis in Animalia [8]. Although the gene *Pax6* is involved in eye morphogenesis of insects and vertebrates, their eye morphogenesis GRNs differ and neither network is conserved across Bilateria, the smallest group that includes both insects and vertebrates. *Pax6 *may have predated the Bilateria and have been independently co-opted into the different GRNs for eye morphogenesis in insects and vertebrates (Fig. **1**). There is no eye morphogenesis ChIN for the Bilateria, the smallest group that includes both insects and vertebrates, but there may be an eye morphogenesis ChIN for vertebrates and another one for insects (recall that rigorous statements of homology specify the phylogenetic level of homology or conservation; here Bilateria, Vertebrata or Arthropoda). 

Wagner uses phylogenetics to determine which gene networks are homologous and which gene networks have been independently assembled, as in the case of eye morphogenesis networks in insects and vertebrates. Thus, even among proponents of biological homology, common ancestry is still the metric by which homology is measured: “characters found in different species are homologous if they are derived from the same character in their most recent common ancestor” [8]. To understand the evolution of segmentation, we need to know at what phylogenetic level the segment polarity GRN is conserved: is the segment polarity GRN a homology of insects or arthropods or bilaterians? Did the segment polarity GRN occur in the common ancestor of vertebrates and arthropods or does this GRN underlie only arthropod segmentation? If the segment polarity GRN predates arthropods, it is clearly not an arthropod segmentation ChIN.* In order to identify a ChIN, a gene network must first be validated as a taxic homology and it must be homologous at the same phylogenetic level of inclusiveness as the phenotypic trait it is hypothesized to underlie.*

### 4.2. ChINs Can Illuminate Phenotypic Transformation in a Comparative Context

Reconstructing the tree of life through analyses of character distributions (whether molecular or morphological) across taxa has been the focus of phylogenetic research. In this process, taxic homologies (whether molecular or morphological) are the primary data. However, there is another kind of homology, transformational homology, which has been outside the purview of phylogenetic analysis but has sometimes been regarded as the most interesting kind of homology, even by phylogeneticists [4].

**Fig. (3):**
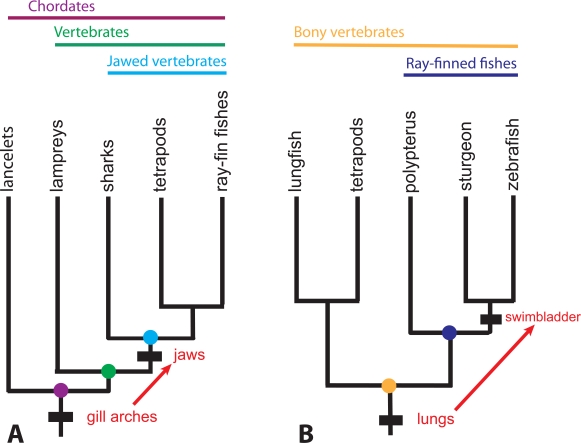
**Two highly pruned phylogenetic trees showing four taxic homologies (gill arches, jaws, lungs, swimbladders) and two transformational relationships between taxic homologies. A**. Phylogeny of Chordates, with two taxic homologies (gill arches and jaws) mapped. The hypothesized transformation of gill arches to jaws is shown by a red arrow. Evidence for this transformational hypothesis is from anatomy, histology, and gene expression. **B**. Phylogeny of bony vertebrates with two taxic homologies (lungs and swimbladder) mapped. The hypothesized transformation of lungs to swimbladder is shown by a red arrow. Evidence for this transformational hypothesis is from anatomy, histology, and gene expression.

Transformational homology pertains to structures sufficiently modified from the ancestral condition that the unmodified and modified structures are seen as having somewhat different identities. Jaws are thought to be transformational homologues of gill arches; limbs are transformational homologues of fins; the bones of the mammalian middle ear are transformational homologues of elements of the jaw articulation of basal lobe-finned fishes. The swim bladder is thought to be a transformational homologue of lungs. Transformational homologies do not explicitly define groups the way taxic homologies define groups, but versions of them can define subgroups: gill arches are a taxic homology of chordates; nested within chordates, jaws are a taxic homology of jawed vertebrates (gnathostomes). “Jaws are modified gill arches” is a hypothesis of transformational homology (Fig. **3**).

Transformational homology has been seen as intractable by phylogeneticists; the methods of phylogenetic analysis can identify only taxic homologues, but not confirm transformational homology [4]. Phylogenetic analysis can validate jaws as a homology of jawed vertebrates (gnathostomes) and gill arches as a homology of chordates, but demonstrating that jaws are modified gill arches requires other information such as comparative anatomy or comparative developmental genetics. Even so, phylogenies still provide context. Phylogenetics tells us that the distribution of a modified structure is nested within the group defined by the unmodified structure and thus establishes the direction of transformation. Jaws are modified gill arches and swimbladders are modified lungs, not the reverse (Fig. **3**).

Comparative anatomists have hypothesized a number of transformational homologues, but controversy increases the more a hypothesized transformational homologue differs from its purported antecedent structure. While there is little disagreement that vertebrate jaws are modified gill arches and that the bones of the middle ear are modified elements of the jaw articulation of fleshy-finned fishes, there is greater controversy about other transformational homologies. For example, paired appendages of vertebrates have been hypothesized to be modified gill rays or modified fin folds [18]. Swimbladders of ray-finned fishes have been hypothesized either to be modified lungs [19-21] or independent outpocketings of the pharynx [22]. In the latter case, lungs are a homology of bony vertebrates [23] and the swimbladder is a homology of a subset of the ray-finned fishes, the Actinopteri [24], but there is no phylogenetic method that can substantiate that swimbladders are modified lungs rather than independent outpocketings of the gut (see Fig. **3**). Nevertheless, lungs and swimbladders (collectively air-filled organs) have been regarded as transformational homologues for more than a century [21, 24] based on anatomical and histological evidence [19, 21]. Phylogenetics uses the variable distributions of characters among taxa to reconstruct the nested relationships of taxa within taxa. Phylogenetics does not address how the characters themselves have been transformed through nested sets of taxa. 

Study of gene networks, specifically ChINs, can illuminate the process of phenotypic transformation in several ways. First, identification of a ChIN underlying hypothesized transformational homologues can substantiate that one structure is a modified form of another structure. Second, in concert with phenotypic and phylogenetic data, ChINs can be used decipher the relationship between genotype and phenotype. For example, a GRN found in all lineages of bony vertebrates is a taxic homology for bony vertebrates (Osteichthyes). If that GRN is critical for the development of lungs and swimbladders *but not for the development of other structures*, this would constitute evidence that the GRN is a lung-swimbladder ChIN and that the lung and swimbladder are transformational homologues. As long as there is a kernel of a ChIN in both hypothesized transformational homologues (and there doesn’t have to be; see below) we can recognize transformational homology. 

For a GRN to be a ChIN, the network must be conserved across taxa in the relevant group or clade (congruent with other homologous characters). For a ChIN to substantiate a hypothesized transformational relationship, the hypothesized ChIN must be involved in specification of both hypothesized transformational homologues, but not be involved in the specification of other structures. Regulatory network modules that operate in multiple unrelated processes (e.g. signaling cascades) and structures would not be useful for recognizing transformational homology. Such widely deployed GRNs are presumably ancient, conserved at deep phylogenetic levels, and have been co-opted independently in a variety of developmental contexts. GRNs that *can* be informative as ChINs must be deployed in relatively limited contexts, or include unique network interactions found only in a particular organ system. For example, in mouse, expression of *Nkx2.1* is known only in telencephalon, thyroid, and lungs, and in each context it may be part of a different GRN. Thus, the transformational relationship between lungs and swimbladders can be tested by examining whether the network which includes *Nkx2.1* expression in mouse lungs is also deployed in zebrafish swimbladder development (Cass *et al., *submitted).

### 4.3. Deep Homologies of Molecular and Cellular Building Blocks Reveal the Assembly of Phenotypic Novelties While ChINs can Substantiate Phenotypic Transformation

A complex trait or novelty includes both the phenotype and the underlying gene networks that specify that phenotype. Any component of a complex trait, whether genotypic or phenotypic, can evolve without rendering the trait unrecognizable. Different aspects of phenotype evolve at different rates. Different aspects of underlying genetics also evolve at different rates. A change in a gene network does not always result in a change in the specified phenotype. For example, the notochord is an unambiguous homology of chordates (notochords characterize chordates), but recent work has shown that there is divergence in notochord development at the molecular developmental genetic level [25, 26]. A change in morphology may not be a consequence of a change in an underlying ChIN, but rather a consequence of other altered genetics, altered embryonic context or epigenetics. It seems conceivable that over time (enough), an underlying ChIN could evolve beyond recognition, but for transformed morphological traits where an underlying ChIN is still intact, we can use ChINs to identify transformational homologues. ChINs are simply the most conserved parts of networks that specify essential aspects of morphological traits. If we think of morphological traits as the product of interacting genes, molecules, cells, and tissues, there is no reason to expect that aspects of morphology are any more or less likely to evolve than aspects of the interacting components that produce a particular phenotype. Hence, the same morphologies can have different genetic developmental bases as in notochords [25, 26]. Different morphologies,as exemplified by gill arches and jaws, can have a substantially similar developmental genetic basis. ChINs provide evidence for such transformed morphologies. 

**Fig. (4):**
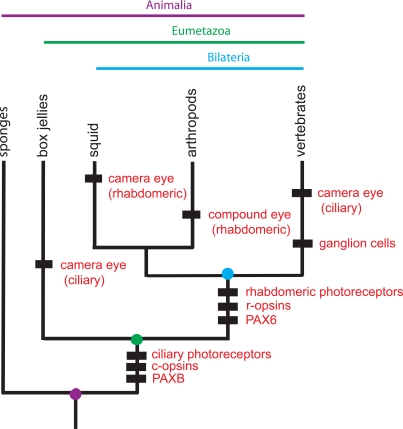
**Homology (deep homology) and non-homology in eye evolution.** Camera eyes of vertebrates and squid are not homologous; that is, they have evolved independently. However, *Pax6*, which is homologous for the more inclusive (deeper level of) Bilateria, has been co-opted independently in eye development of both squid and vertebrates. Rhabdomeric photoreceptors and r-opsins are also homologous for Bilateria. Vertebrate ganglion cells have been hypothesized to be a transformational homology of rhabdomeric photoreceptors. Camera eyes of box jellies are thought to be derived independently from those of vertebrates. The figure is modified from Shubin *et al.* [10].

Great strides have been made in understanding the molecular development of a number of phenotypic novelties, such as segments, eyes or limbs. Understanding the evolution of these novelties requires exposing the molecular development of the same trait in a comparative context, that is, among many phylogenetically related non-model organisms. By identifying the specific phylogenetic levels at which various molecular and cellular components of a phenotypic trait arise, we can unravel the process by which genes, networks and cell types (various deep homologies) have been co-opted to build such evolutionary novelties (Fig. **4**) [10]. Modern genomic methods are making it possible to expose the genetic basis of traits of interest in any and many non-model organisms. When coupled with advanced methods for manipulating genes (e.g., siRNA; morpholinos; transgenesis; zinc finger nuclease disruptions), hypotheses regarding conserved functions of certain genes can be tested experimentally, both with respect to phenotypic outcome and impact on presumed GRNs.

## 5. THE CONTRIBUTION OF MODERN COMPARATIVE AND FUNCTIONAL GENOMICS TO UNDER-STANDING PHENOTYPIC TRANSFORMATION IN AN EVOLUTIONARY FRAMEWORK

Developmental genetics and functional genomics determine whether a GRN is involved in the development of a structure, and whether a ChIN of a particular morphology is involved in both the unmodified and hypothesized transformational homologue. New genomic approaches to generate whole genomes of non-model organisms, will facilitate characterization of gene networks, essential to the character of a morphological trait (ChINs).

In a comparative context, breathtaking advances in genomic technologies are removing practical barriers to obtaining comprehensive genomic and molecular information on any species. For many years, the major barrier to high resolution systematic studies was the ability to obtain primary sequence of a “new” genome for a non-model taxon. This required generation of genomic libraries (made in bacteriophage or Bacterial Artifical Chromosomes vectors, for example), followed by (usually) high throughput strategies for creating physical maps that could be integrated with genetic maps. Indeed, this approach was used for the earliest sequenced large genomes such as mice and humans, and is still valuable today for ensuring the completeness and correct assembly of contiguous sequence throughout a genome. This strategy was replaced by whole genome shotgun (WGS) strategies pioneered by Craig Venter in flies and humans, in which cloned DNA molecules of defined length were end-sequenced using standard, but highly automated Sanger sequencing methods yielding long read lengths (several hundred bps) [27]. While read lengths were long, the number of “reads”, or individual sequencing reactions that can be performed by this technology was rather limited, both by the sequencing chemistry and instrument technology that required individually prepared DNA templates.

All this has changed with the availability of “Next Generation” DNA sequencers. At present, the three major platforms (made by Illumina, Applied Biosystems, and Roche) have in common one major difference with traditional DNA sequencing: there are no cloning steps [28]. Although a “library” is prepared as the starting material for sequencing, such libraries are prepared in single tubes and simply represent a large number of DNA molecules that have proper oligonucleotide adaptors that are required for the DNA sequencing reactions in the instrument, each of which originates from a single molecule in the library, but which is usually amplified by a PCR-like reaction. In this sense, the library is a misleading term, since the exact templates in the library are unknown and cannot be retrieved once they are used. Although the Roche instrument can generate reads of a few hundred bp, the Illumina instrument produces many more DNA sequence reads of much shorter length (~100bp) from a single run (at present, at a rate of up to 25 Gb/day, which is nearly 10X the size of the human genome).

Enabled by this high sequencing throughput capability, it has become routine to “re-sequence” genomes, particularly the human. That is, the sequence data generated is aligned with the consensus, (essentially) complete and contiguous genome sequence. This is routine and relatively simple. Such experiments are done to find mutations, genetic variation or genome rearrangements between people. It is a universal goal for the cost of resequencing a human genome to decrease to $1,000 in the near future. However, it is far more difficult and expensive to generate a complete and contiguous genome sequence of a “new” genome, and this is complicated by the relatively short sequence reads of the Illumina and Roche sequencers, and also the relatively high error rate that complicates proper assignment of repetitive or duplicated sequences to the proper places in the genome. Nevertheless, “Next-gen” sequencing has been used recently to generate de novo genomic sequence of a mammal, the Giant Panda, using Illumina instruments [29]. This feat was neither trivial nor cheap, and gaps remain to the drawback of short reads. The genomes of other organisms have also been assembled de novo using Next-gen sequencing, although none of these assembled genomes are “complete” by any means [30].

The problems associated with creating full and accurate de novo genome sequences is probably short-lived due to the impending deployment of a third generation of DNA sequencers with enormous potential. Of particular relevance for sequencing of new genomes is the single molecule sequencing platform manufactured by Pacific Biosciences. Not only are reagent costs low and sequence runs short (under 1 hour), but most importantly, these machines can generate extremely long reads of several kilobases [31]. Long read capability will overcome many of the obstacles to assembling genomic sequence contiguously along chromosomes, since the read length exceeds the size of long interspersed repeat elements. In short, the barriers to *de novo* whole genome sequence assemblies are crumbling, and this enables higher order comparative genomics of diverse non-model species.

The ability to generate contiguous genome assemblies quickly and cheaply, even with a substantial error rate in the primary DNA sequence, is critical to overlaying gene information, particularly gene expression data. The “RNA-Seq” method of generating transcriptome information is particularly suited to expression profiling newly sequenced genomes [32]. First, the need for making traditional cDNA libraries is overcome. Second, no information about the target genome is needed to generate the data, and thus microarrays are not required. However, the short reads (especially of genes expressed at low levels) from RNA-Seq experiments (which generally are performed most commonly on the Illumina platform) can confound assembly of full-length transcripts in a “new” genome. Having a genome assembly helps overcome this problem by enabling assignment of RNA-seq reads to genomic locations, thus aiding construction of gene models and measurement of relative expression levels. Any tissue or structure of interest can be subjected to RNA-seq-based gene expression profiling, and minimal sample amounts are needed. The marriage of third-generation de novo genome assembly with RNA-Seq expression profiling thus enables one to address specific questions pertaining to the functional genomic basis of phenotypic evolution.

An important benefit of having a genome sequence and RNA-seq data is that the resulting gene models enable predictions of the proteome. With that in hand, mass spectrometry can be used to examine proteome-wide post-translational modifications (e.g. protein phosphorylation, ubiquitination, acetylation, sumoylation, etc.) in an organism under various physiological or environmental conditions. This could yield important functional insights into the similarities and differences between homologous molecular networks across species.

Probably the greatest roadblock to validating hypotheses about the significance of particular genes in a ChIN is the ability to conduct functional experiments. This depends on two major technical abilities. The first is the biology of the organism, and whether it can be experimentally manipulated and propagated in the laboratory. This points out the potential need to make a new series of strategically-placed model organisms that are amenable to relatively easy husbandry. The second obstacle is the ability to genetically manipulate the organism to query the function of individual genes, possibly with the goal of mimicking putative evolutionary modifications.

Since the advent of recombinant DNA technology, the ability to modify the genome of a species has become a major determinant of whether that species becomes a viable model organism. More recently, the ability to mutate specific genes has become a crucial feature. In mice, these abilities were enabled by DNA pronuclear microinjection for transgenes, and ES cell knockouts for making custom mutations. In *C. elegans*, there is facile transgenesis for adding genes, and although it is not easy to selectively knock out genes, this organism is easily subjected to whole-body siRNA-mediated knockdown of any gene [33]. Zebrafish fall somewhere in between; transgenic technology is routine, but knockouts are not. Alternatively, expression of genes can be knocked down using modified “morpholino” oligonucleotides. However, the target gene must be expressed early in development, because these modified oligonucleotides are injected into early stage fertilized embryos, and they become diluted over time. However, even this shortcoming can be overcome using strategies such as creating transgenic lines expressing short hairpin RNAs (shRNAs) in a cell type of choice, and even in inducible manners. For new organisms in which transgenesis has not been developed, it may be more practical to infect tissues of interest by injecting viral vectors containing shRNAs transcribed by generic promoters.

Finally, for an organism in which transgenesis is possible, or where embryos or oocytes can be manipulated in the lab, zinc finger nuclease (ZFN) technology has emerged as a powerful and universal technology for manipulating the germline of an organism [34]. Armed with genomic sequence, this technology involves modular synthesis of a zinc finger nuclease (ZFN) enzyme custom selected to bind and cleave an unique sequence in the genome. The creation of a double stranded break is universally recombinogenic; therefore, when the ZFN is co-introduced into the embryo/oocyte with a DNA construct with sequence identity to the DNA that is cleaved, there is efficient recombination that results in the incorporation of the foreign construct into the genome. This construct can be designed to mutate the cognate host gene, and perhaps even include a reporter (such as GFP).

## 6. CONCLUSIONS

The terminology relating to homology has a long confusing history of controversy and debate. However, a simple phylogenetic view of homology, where homology has been recognized as simply a trait characterizing a natural group (i.e. a synapomorphy [such as vertebrae] characterizes a monophyletic group, [such as the Vertebrata]) provides a rigorous framework for comparative study of molecular developmental genetics in an evolutionary context.

Homology is simply similarity due to common ancestry and it is synonomous with taxic homology and synapomorphy. Continuity of genetic information (“biological homology”) is the reason for homology, not homology itself. Gene networks conserved for two traits, one putatively modified from another (e.g. jaws from gill arches or limbs from fins) can provide evidence for transformational homology. All “deep homologies” are taxic homologies, but when deep homologies are the molecular or cellular building blocks deployed in relatively recently evolved phenotypic novelties, they have particular evolutionary interest. Mapping deep homologies of molecular and cellular components a phenotypic trait on a phylogeny can reveal the sequence by which complex novelties evolve.

Comparative molecular evolutionary development has been previously limited by the lack of reference genomes for non-model taxa. New developments in comparative genomic technology will stimulate an explosion of comparative genomic data for non-model organisms, enabling sophisticated phylogenetic analyses of the molecular genetic basis for important phenotypic novelties that characterize evolutionary history. It is with rigorous mapping of these molecular genetic homologies in a phylogenetic framework that will allow us to reconstruct the steps by which complex novelties have evolved.
